# Risk Assessment of RNAi-Based Potential Pesticide ds*NlAtg3* and Its Homologues for *Nilaparvata lugens* and Non-Target Organisms

**DOI:** 10.3390/insects16020225

**Published:** 2025-02-19

**Authors:** Kai Li, Tongtong Chen, Yuliang Li, Kai Sun, Kun Pang, Xiaoping Yu, Peiying Hao

**Affiliations:** Key Laboratory of Microbiological Metrology, Measurement & Bio-Product Quality Security, State Administration for Market Regulation, China Jiliang University, Hangzhou 310018, China; 17816121215@163.com (K.L.); nadinechentt@163.com (T.C.); 18853663892@163.com (Y.L.); sunkai@cjlu.edu.cn (K.S.); pangk@cjlu.edu.cn (K.P.)

**Keywords:** RNA interference, *Nilaparvata lugens*, non-target organism, natural enemy, biosafety

## Abstract

The brown planthopper (*Nilaparvata lugens*) is an insect pest of rice, which mainly feeds on the phloem sap of the leaf sheath. RNA interference (RNAi) has application prospects in pest control, but it is necessary to select target genes and design suitable dsRNA fragments for RNAi so that it can achieve effective pest control and avoid risks to non-target organisms. NlAtg3 is a key protein in the autophagy pathway of *N. lugens*. Three kinds of dsRNA fragments of the *NlAtg3* gene (ds*NlAtg3-*474×1, ds*NlAtg3*-138×3 and ds*NlAtg3*-47×10) were designed to compare the RNAi efficiency and specificity against the target insect *N. lugens* and non-target organisms through microinjection. The results showed that the fragment ds*NlAtg3-*474×1 showed strong inhibitory effects on the survival of *N. lugens*, which resulted in the survival rate decreasing to zero on the fifth day, while the survival rate of a closely related species, *Sogatella furcifera*, dropped to 2.22%. In contrast, ds*NlAtg3*-47×10 specifically designed against *N. lugens* only showed slight or no inhibitory effects on *S. furcifera* and other non-target organisms such as *Drosophila melanogaster*, but still showed good lethal effects against *N. lugens*, with the survival rate dropping to 18.89% on the ninth day. In addition, after being fed *N. lugens* injected with ds*NlAtg3*-47×10 fragments, the survival rate of the natural enemies *Dolomedes sulfureus* and *Tytthus chinensis* did not show significant change, compared with those treated with the ds*GFP* control. Our results suggest that the *NlAtg3* gene can serve as a potential target for controlling *N. lugens*. Moreover, by designing suitable RNAi fragments, it is possible to avoid harm to non-target organisms while effectively inhibiting the target insect *N. lugens*.

## Simple Summary

The brown planthopper (*Nilaparvata lugens*) is a serious pest insect of rice. RNA interference technology for controlling *N. lugens* has good prospects based on its advantages, such as high species specificity, low potential for environmental pollution, and obvious effects. However, it is necessary to select suitable target genes and design species-specific dsRNA fragments for RNAi so that it can achieve effective pest control and avoid the risk to non-target organisms. In this study, we evaluated three different RNA interfering fragments targeting an autophagy-related gene *Atg3* (ds*NlAtg3-*474×1, ds*NlAtg3*-138×3 and ds*NlAtg3*-47×10) of *N. lugens* and also assessed their effects on non-target species. The results showed that all three ds*NlAtg3* fragments were effective against *N. lugens*. In addition, the effects of ds*NlAtg3*-47×10 (specifically designed against *N. lugens*) on another four organisms, including two natural enemies, *Dolomedes sulfureus* and *Tytthus chinensis*, were tested. The results showed that the fragment ds*NlAtg3*-47×10 had no significant effect on the survival or development of non-target organisms. Therefore, the fragment ds*NlAtg3*-47×10 has good potential to be developed as insecticide for controlling *N. lugens*.

## 1. Introduction

The brown planthopper *Nilaparvata lugens* is an insect pest of rice [1]. *N. lugens* damages rice by sucking the phloem sap of the leaf sheath, laying eggs in rice tissues, and transferring a variety of rice viruses [2,3]. However, with the misuse of insecticides, *N. lugens* has developed resistance to chemical insecticides [4,5]. Breeding new insect-resistant rice varieties is an environmentally friendly method, but *N. lugens* feeding on an insect-resistant rice variety for several generations can generally adapt to this variety. RNA interference (RNAi) can inhibit mRNA expression levels of the target gene in organisms, including in some species of insect. Therefore, clarifying gene function and precisely manipulating specific gene expression in insects [6,7] has opened a new pathway for effective control of insect pests.

RNAi-based insect-resistant genetically engineered (IRGE) crops have shown good application prospects in pest control. In some corn varieties, RNAi via the dsRNA of *DvvSnf7*, *Cry3Bb1*, and *Cry34Ab1*/*Cry35Ab1* genes caused significant death of western corn rootworms [8,9]. Currently, transgenic rice expressing dsRNA has made significant breakthroughs in the management of *N. lugens*. Rice expressing dsRNA of the *Nlsid-1* and *Nlaub* genes of *N. lugens* has been demonstrated to inhibit the expression of target genes in the midgut [10]. It has been reported that rice plants with overexpression of *osa-miR162a* had enhanced resistance against *N. lugens* and reduced the reproductive capacity of adults [11]. However, some rice-mediated RNAi had little effect on the survival rate of brown planthopper. It has been reported that the expression of *NlGST1*-*1* and *GST* activity in *N. lugens* nymphs was significantly inhibited after the insects fed on transgenic rice expressing *NlGST1*-*1* dsRNA, but the lethal phenotype of *N. lugens* nymphs after feeding was hardly observed [12]. It generally takes a long time to acquire insect-resistant genetically engineered rice, so before constructing IRGE rice, it is necessary to screen suitable target genes for RNAi first through convenient methods like microinjection of dsRNA or feeding-based RNAi methods.

In addition to the selection of the target gene, the effect of RNAi is also affected by several other factors, including the length, the sequence composition, and the concentration of dsRNA fragments [13,14]. After entering the cells of an insect, dsRNA fragments are first cleaved into siRNA by Dicer-2 enzyme or Dicer-2 nuclease homologues. Fragments of siRNA are usually 19–24 nt, and their bases at the 3′ end protruding from the sticky end and the phosphate group at the 5′ end play key roles in their functions. However, when Dicer-2 enzymes in insects cleave exogenous dsRNA, short fragments of varying length or sequence are produced due to the irregular cleaving position, thus affecting RNAi efficiency [15,16]. Moreover, the dsRNA length for inducing effective RNAi varies among different insects [17]. In *Drosophila melanogaster* S2-cells, dsRNAs ranging from 21 to 592 bp in length all resulted in effective silencing of the target gene, irrespective of the dsRNA length, after being forcibly introduced into cells by transfection. In contrast, uptake of dsRNAs added to the medium was clearly length-dependent [18].

For insects, autophagy is essential for growth and development. Some studies have shown that the loss of *Atg9* leads to a shortened lifespan, motor deficits, and increased susceptibility to stress in *Drosophila*. *Atg9* loss also resulted in aberrant adult midgut morphology with dramatically enlarged enterocytes [19]. Abdul indicated that LAMP2A can potentiate autophagic flux in the *Drosophila* brain, leading to enhanced stress resistance and neuroprotection [20]. *ATG3* is one of the key genes in the autophagy pathway, and encodes an E2 ubiquitin-like binding protein in the ATG2 system, promoting the extension of phagophore [21]. Our previous study showed that RNAi through microinjection of *dsNlAtg3* into the fifth-instar nymphs of *N. lugens* resulted in a perfect lethal effect, indicating its possible role in pest control [22]. However, the minimum effective length of a dsRNA fragment specific to *N. lugens*, and its effect on non-target organisms including natural enemies, are currently unavailable. Therefore, it is necessary to screen suitable dsRNA fragments of *ATG3* specific to *N. lugens* and explore their effects on non-target organisms for the construction of a rice-mediated RNAi system for controlling *N. lugens* in the future.

## 2. Materials and Methods

### 2.1. Insects

The experimental insects of *N. lugens* and *Sogatella furcifera* have been reared on TN1 rice over ten years. The *Dolomedes sulfureus* (Araneae: Pisauridae) was collected from the rice paddy of China Jiliang University, Hangzhou, China. The *Tytthus chinensis* (Heteroptera: Miridae) was collected from a rice paddy in Ningbo, China. The *Drosophila melanogaster* was purchased from the Qidong Fungene Biotechnology company (Qidong, JiangSu, China) and fed with a medium consisting of agar, yeast and cornmeal. The insects and rice plants were maintained at a temperature of 26 ± 2 °C, 75% ± 5% RH, and a photoperiod of 16L:8D.

### 2.2. Double-Stranded RNA Synthesis and Microinjection

**Figure 1:**
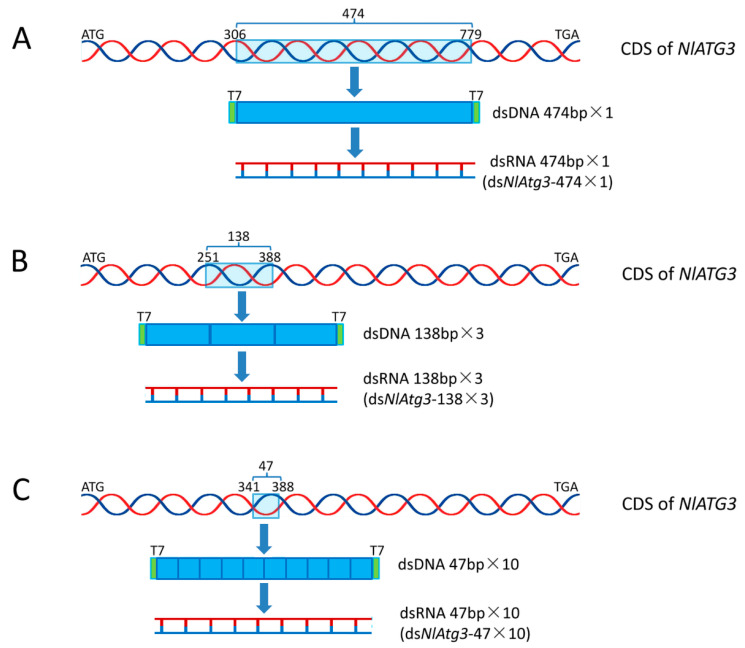
Schematic diagram of the location and repetition of different dsRNA fragments against *NlAtg3*. (**A**) Schematic diagram of the 474 bp sequence location selected for synthesizing ds*NlAtg3*-474×1, with the T7 promoter sequence and protective bases added. (**B**) Schematic diagram of the 138 bp sequence location selected for synthesizing ds*NlAtg3*-138×3. The cDNA template used for synthesizing the ds*NlAtg3*-138×3 fragment contains 3 repeats of a 138 bp sequence, T7 promoter and protective bases. (**C**) Schematic diagram of the 47 bp sequence location selected for synthesizing ds*NlAtg3*-47×10. The cDNA template used for synthesizing the ds*NlAtg3*-47×10 fragment contains 10 repeats of a 47 bp sequence, T7 promoter and protective bases.

In order to compare the RNAi efficiency and specification of non-concatemerized long dsRNA (NCL-dsRNA) with concatemerized dsRNAs (C-dsRNAs), three kinds of dsDNA templates (dsDNA-474×1, dsDNA-138×3 and dsDNA-47×10) were prepared accordingly for transcripting NCL-dsRNA (ds*NlAtg3*-474×1) and C-dsRNAs (ds*NlAtg3*-138×3 and ds*NlAtg3*-47×10) against the *NlAtg3* gene (GenBank acc. no.: MF040142.1) from *N. lugens* (Figure 1). Total RNA was extracted and used as the template to synthesize cDNA as previously described [22]. PCR was used to synthesize dsDNA-474×1 using the primers in Supplementary Table S1. dsDNA-138×3 and dsDNA-47×10 (with a *Bam*H I site and T7 promotor at both ends) for synthesizing dsRNA of ds*NlAtg3*-138×3 and ds*NlAtg3*-47×10 were prepared by Personalbio (Shanghai, China) using a chemical synthesis method. For dsDNA-138×3, the repeat unit was a 138 bp sequence, and the whole dsDNA template contained 3 repeats of the 138 bp unit (Figure 1B). Similarly, the whole dsDNA-47×10 template contained 10 repeats of the 47 bp unit. The pMD18-T vector was used to clone the dsDNA products, which were then separately introduced into *E. coli* JM109 and sent to the Ykang company (Hangzhou, China)for sequencing. Following the manufacturer’s instructions, ds*NlAtg3-*474×1, ds*NlAtg3*-138×3 and ds*NlAtg3*-47×10 were then synthesized using the MEGAscript^®^ T7 High Yield Transcription Kit (Figure 1A, Thermo Fisher Scientific, Waltham, MA, USA). *GFP* (GenBank acc. no.: MF169984) was used to synthesize ds*GFP* (1155 bp–1811 bp) as the control. The sequence information of the *Atg3* gene, primers, *Bam*H I site and T7 promotor are listed in Supplementary Table S1.

Microinjection needles were processed by a PC-10 microelectrode tractor (NARISHIGE, Japan, Tokyo). The 5th-instar nymphs of *N. lugens* were injected with 50 nL of dsRNA (250 ng/μL) per insect, using a microinjector (EPPENDORF, Hamburg, Germany). The synthesized dsRNA was injected into the thorax at the site between the middle and hind legs of *N. lugens*. The injected insects were cultured on TN1 rice plants [23].

### 2.3. Effect of RNA Interference on the Survival of N. lugens

To evaluate the effect of different dsRNA fragments on the survival of *N. lugens*, ds*NlAtg3-*474×1, ds*NlAtg3*-138×3 and ds*NlAtg3*-47×10 were injected into the 5th-instar nymphs, and ds*GFP* was used as a control. Each treatment contained 3 replicates, and each replicate pooled 30 nymphs of *N. lugens*. The survival rate and other physiological indicators of *N. lugens* were observed and counted daily.

### 2.4. Gene Expression Analysis Using RT-qPCR

The RT-qPCR technique was performed to analyze the *NlAtg3* expression subsequent to the injection of different dsRNAs. For this purpose, primers (Supplementary Table S1) specific to synthesizing ds*NlAtg3-*474×1-qf and ds*NlAtg3-*474×1-qr were designed with the Primer Premier 5.0 software. The RT-qPCR reaction systems (20 μ) contained 10 μL of TB green RemixExTaqII, forward and reverse primers (10 μM) (0.4 μL), cDNA as a template (2.0 μL), ROX reference dye (0.4 μL), and water (6.8 μL). The PCR reactions were performed using the Step One Plus real-time PCR system (Bio-Rad, Hercules, CA, USA). The reference gene was 18s rRNA (GenBank accession number: JN662398.1) of *N. lugens* (*Nl18S*). Each sample contains 3 technical replicates. The 2^−ΔΔCt^ method was used to detect the relative expression levels of genes [24].

### 2.5. Effect of RNAi on the Survival of Non-Target Organisms

The 5th-instar insects of *S. furcifera* were separately injected with ds*NlAtg3-*474×1, ds*NlAtg3-*138×3 and ds*NlAtg3*-47×10 between 1 and 12 h after molting, and ds*GFP* was introduced by microinjection as a control. Through the thorax, at the site between the middle and hind legs, 250 ng of dsRNA per insect was injected, and the insects were transferred to feed on TN1 rice plants [25]. Each treatment was set up with three duplicates, each consisting of 30 *N. lugens* insects. The survival rates of each group were tracked daily. As a non-target insect, *D. melanogaster* larvae of the third instar were injected with 250 ng of ds*NlAtg3*-47×10 (injected into the larvae’s abdomen as described above) [26]. Prior to injection, *D. melanogaster* was frozen on ice for 5–10 min. The injected larvae were transferred to the Drosophila medium, and their mortality, growth and development were observed and recorded.

### 2.6. Effect of RNAi on Natural Enemies Feeding dsRNA-Treated N. lugens

Three *N. lugens* insects, injected with ds*NlAtg3-*47×10, were first released in a tube, and 24 h later, one *D. sulfureus* was released into the tube to feed these injected *N. lugens*. During the following days, three additional injected *N. lugens* were added to each tube daily. In total, 10 tubes were prepared for ds*NlAtg3-*47×10, and 10 tubes were prepared for ds*GFP* as a control. The mortality of the *D. sulfureus* was observed and recorded every day. *T. chinensis* was treated as described above for *D. sulfureus*.

### 2.7. Statistical Analysis

For gene transcript levels, the data were analyzed by one-way ANOVA followed by Tukey’s multiple comparison test. The comparison of the survival curve was conducted by the Log-rank test. All statistical analyses were conducted on SPSS 22.0 software (http://www.spss.com, accessed on 6 September 2024).

## 3. Results

### 3.1. Spatiotemporal Expression Patterns of NlATG3 Gene in N. lugens

**Figure 2:**
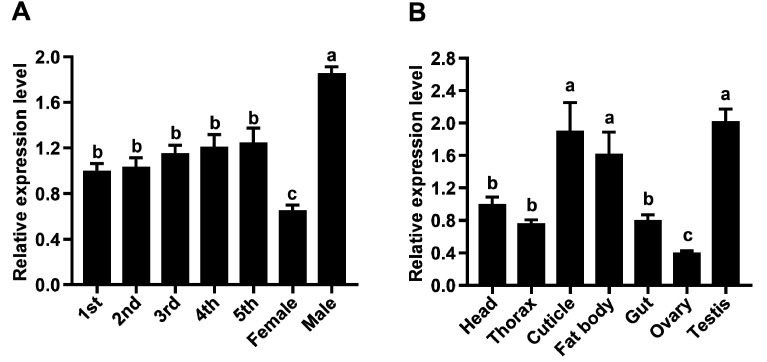
Spatiotemporal expression patterns of the *N. lugens NlATG3* gene. (**A**) Relative expression levels of the *NlATG3* gene at different developmental stages.1st: 1st-instar nymphs; 2nd: 2nd-instar nymphs; 3rd: 3rd-instar nymphs; 4th: 4th-instar nymphs; 5th: 5th-instar nymphs; Female: female adult; Male: male adult. (**B**) Relative expression of *NlATG3* gene in different tissue sites. The data in the figure represent the mean ± SEM of three biological replicates, using one-way ANOVA and multiple comparison test by Tukey’s method, and different lowercase letters over the bars in the figure represent significant differences.

To verify the expression of the *NlATG3* gene in *N. lugens*, its spatial and temporal expression was determined by RT-qPCR. The results showed that *NlATG3* expression was relatively stable in nymphs, but was lower in female adults and significantly higher in male adults (*p* < 0.01) (Figure 2A). The expression level of *NlATG3* was the highest in the testis (2.02), followed by the cuticle (1.92) and the fat body (1.62), while it was the lowest in the ovary (0.40). It was medium in the head (set to 1), thorax (0.77), and gut (0.80) (Figure 2B). Therefore, the *NlATG3* gene was expressed in different tissues or parts at all developmental ages.

### 3.2. Sequence Analysis of dsNlAtg3 Fragments with Different Lengths

**Figure 3:**
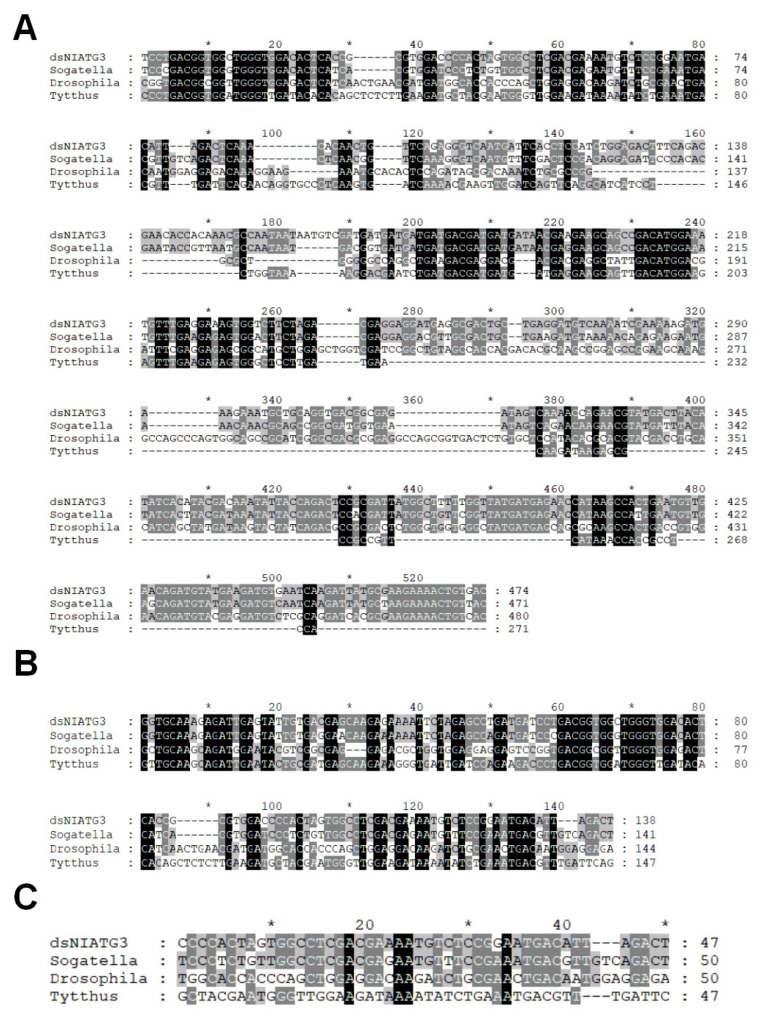
Sequence alignment of different *NlAtg3* fragments with different species. (**A**) ds*NlAtg3*-474×1. (**B**) ds*NlAtg3*-138×3. (**C**) ds*NlAtg3*-47×10. The black color represents that all four sequences contain the same base. The number of identical bases in the four segments gradually decreases, and the color gradually becomes lighter. The *represent every ten-base spacer.

The alignment results showed that ds*NlAtg3*-474×1 of *N. lugens* had 84.07% similarity with that of *S. furcifera*, 51.43% with *D. melanogaster*, and 35.17% with *T. chinensis*. For ds*NlAtg3*-474×1 of *N. lugens*, the longest consecutive identical bases were 27, 16, and 13 in *S. furcifera*, *D. melanogaster*, and *T. chinensis*, respectively (Figure 3A). The ds*NlAtg3*-138×3 of *N. lugens* had 86.52% similarity with that *S. furcifera*, 55.78% with *D. melanogaster*, and 65.28% with *T. chinensis*, respectively. For ds*NlAtg3*-138×3 of *N. lugens*, the longest consecutive identical bases were 25, 8, and 13 in *S. furcifera*, *D. melanogaster*, and *T. chinensis*, respectively (Figure 3B). ds*NlAtg3*-47×10 of *N. lugens* had 78% similarity with that of *S. furcifera*, 32% with *D. melanogaster*, and 40% with *T. chinensis*, respectively. For ds*NlAtg3*-47×10 of *N. lugens*, the longest consecutive identical bases were 12, 5, and 3 in *S. furcifera*, *D. melanogaster*, and *T. chinensis*, respectively (Figure 3C). The sequence of *Atg3* in *D. sulfureus* is not yet available, and is not included in the alignment. However, low conservation is expected between *D. sulfureus* and *N. lugens*, taking *Atg3*-47×10 into consideration. As a spider, *D. sulfureus* has a relatively distant genetic relationship with the insect *N. lugens*, compared with the other insects *S. furcifera*, *D. melanogaster*, and *T. chinensis*. By reducing the longest consecutive identical sequence in dsRNA sequence, it is possible to avoid harm to non-target organisms.

### 3.3. RNAi Efficiency of Different dsRNA Fragments on the Expression of NlAtg3 in N. lugens

**Figure 4:**
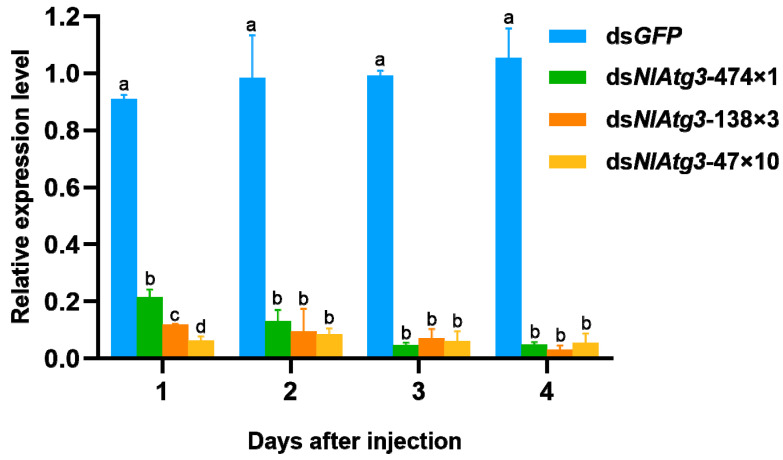
Expression levels of *NlAtg3* mRNA in the 5th-instar nymphs of *N. lugens* after injection of different kinds of interference fragments. ds*GFP* was used as the control group, and Nl*18s* was used as the internal reference gene for gene expression normalization. n = 5 insects. Bars are the mean ± SEM from three independent experiments. The data were analyzed by one-way ANOVA followed by Tukey’s multiple comparison test. Different lowercase letters on the bars indicate significant differences (*p* < 0.05).

The effect of RNAi on the expression of the target gene *NlAtg3* was analyzed by injecting fifth-instar *N. lugens* nymphs with 250 ng of dsRNA. RT-qPCR revealed that the mRNA expression of *NlAtg3* in all treated groups was significantly suppressed compared with the ds*GFP* control. On day 1, treatment with ds*NlAtg3*-474×1, ds*NlAtg3*-138×3, and ds*NlAtg3*-47×10 resulted in large decreases in the mRNA levels of 78.5%, 88.2%, and 93.7%, respectively (Figure 4). It also showed that dsRNA containing more concatemerized repeats had a better inhibitory effect at this stage, that is, ds*NlAtg3*-47×10 > ds*NlAtg3*-138×3 > ds*NlAtg3*-474×1. From day 2 on, the mRNA expression of the three treated groups did not show significant differences between different treatments, but dsRNA containing more ds*NlAtg3* repeats still showed a better inhibitory effect than that containing fewer repeats on day 2. On day 4 post injection, the expression of the target gene in all treated groups remained at very low levels; treatment with ds*NlAtg3*-474×1, ds*NlAtg3*-138×3, and ds*NlAtg3*-47×10 decreased the expression by 95.33%, 97.09%, and 94.84%, respectively. These results showed that the expression of the target *NlAtg3* gene in *N. lugens* was successfully knocked down through RNAi.

### 3.4. Effect of Different dsRNA Fragments on the Survival of N. lugens

**Figure 5:**
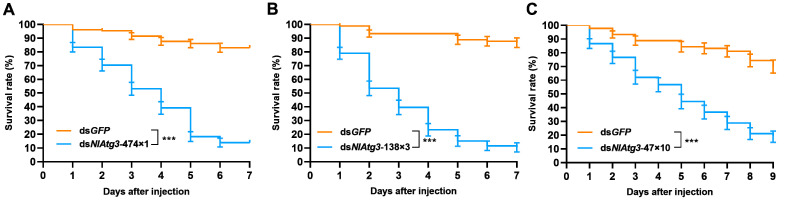
Effect of injecting different lengths of dsRNA fragments on the survival rate of *N. lugens*. (**A**–**C**) Effect of injecting ds*NlAtg3*-474×1, ds*NlAtg3*-138×3 and ds*NlAtg3*-47×10 on the survival rate of *N. lugens*. The comparison of the survival curve was conducted by the Log-rank test. Error bars indicate the standard error (SE) from three independent experiments, with n = 30 insects per treatment. Histogram bars annotated with asterisks are significantly different (***, *p* < 0.001). Significant differences between means (multiple *t*-tests) are depicted.

The fifth-instar *N. lugens* larvae were separately injected with ds*NlAtg3-*474×1, ds*NlAtg3*-138×3, or ds*NlAtg3*-47×10, at 250 ng per insect. In the ds*NlAtg3-*474×1 group, nymph survival decreased significantly and the survival rate decreased to 13.91% on day 7, while the survival rate of the ds*GFP* control group still remained at a high level of 93.33% on day 4 (Figure 5A). After injection with ds*NlAtg3*-138×3, the survival rate decreased from day 1, and decreased to 10.51% on day 7 (Figure 5B). A strong effect was also achieved in nymphs injected with ds*NlAtg3*-47×10, with the survival rate reaching 18.89% on day 9. In contrast, the survival rate of the ds*GFP* control group decreased relatively slowly, to 70% on day 9 post injection (Figure 5C). This indicates that the length of the dsRNA fragments was related to the effect of RNAi on the survival of *N. lugens*.

### 3.5. Effects of Different dsNlAtg3 Fragments on the Closely Related Species S. furcifera

**Figure 6:**
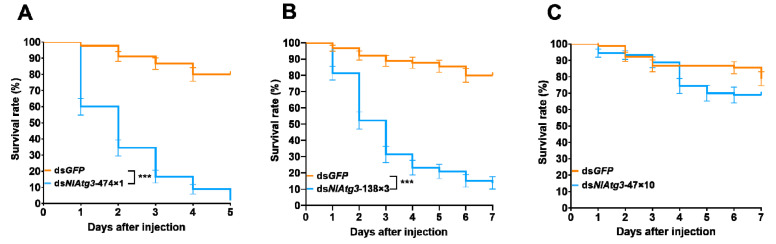
Effects of injecting ds*NlAtg3* fragments of different lengths on the survival rate of *S. furcifera*. (**A**) Effect of injecting ds*NlAtg3-*474×1 on the survival rate of white-back planthopperds. (**B**) Effect of injecting ds*NlAtg3*-137×3 on the survival rate of *S. furcifera*. (**C**) Effect of injecting ds*NlAtg3*-47×10 on the survival rate of *S. furcifera*. Injection of ds*GFP* is used for controls. The comparison of the survival curve was conducted by the Log-rank test. Error bars indicate the standard error (SE) from three independent experiments, with n = 30 per treatment. Histogram bars annotated with asterisks are significantly different (***, *p* < 0.001); no asterisks indicate no significant differences among the comparisons.

*S. furcifera* and *N. lugens* belong to the same family (Delphacidae). By injecting 250 ng of ds*NlAtg3*-474×1, ds*NlAtg3*-138×3, and ds*NlAtg3*-47×10 into each fifth-instar nymph of *S. furcifera*, the impact of different dsRNA fragments on the survival of *S. furcifera* was investigated. The results showed that these three kinds of dsRNA fragments had different effects on the survival rate of *S. furcifera*. After injecting ds*NlAtg3*-474×1, the survival rate declined rapidly to 2.22% on the fifth day post injection. Meanwhile, the survival rate of the ds*GFP* group remained at a high level of 79.26% (Figure 6A). The treatment of ds*NlAtg3*-138×3 achieved similar effects to that of ds*NlAtg3*-474×1 (Figure 6B). Nonetheless, no significant difference in the survival rate was observed between the ds*NlAtg3*-47×10 group (68.89%) and the ds*GFP* control group (78.89%) on day 7 (Figure 6C). This indicates that by designing suitable RNA interference fragments, it is possible to avoid harm to closely related species while effectively inhibiting the target pest *N. lugens*.

### 3.6. Effects of Short Concatemerized dsNlAtg3-47×10 on Non-Target Organism D. melanogaster

**Figure 7:**
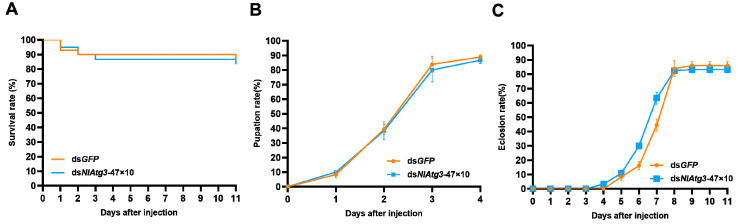
Effects of ds*NlAtg3*-47×10 injection on fly survival and growth and development. (**A**) Survival rate. (**B**) Pupation rate. (**C**) Eclosion rate. Injection of dsGFP used as controls. The comparison of the survival curve was conducted by the Log-rank test. Error bars indicate the standard error (SE) from three independent experiments, with n = 20 per treatment. No asterisks indicate no significant differences among the comparisons.

*D. melanogaster* belongs to another genus of insects, *Drosophila*, and has a relatively distant evolutionary relationship with *N. lugens*, compared with *S. furcifera*. After injection of ds*NlAtg3*-47×10, there was no substantial reduction in the survival rate of *Drosophila* (83.33%) compared to the dsGFP control group (86.11%) on the 11th day post injection (Figure 7A). On day 4 after ds*NlAtg3*-47×10 injections, the pupation rate of *Drosophila* reached 86.67%, and it was not significantly different from that of the control group injected with ds*GFP* (Figure 7B). On day 11 after injection of ds*NlAtg3*-47×10, the pupation rate of *D. melanogaster* reached 83.33%, while the pupation rate of its control group ds*GFP* was 86.11%, with no significant difference observed between the two groups in terms of survival rate (Figure 7C). These results suggest that ds*NlAtg3*-47×10 targeted against *N. lugens* had no significant effect on the growth and development of the non-target organism *D. melanogaster*.

### 3.7. Effects of dsNlAtg3-47×10 on Natural Enemies of N. lugens

**Figure 8:**
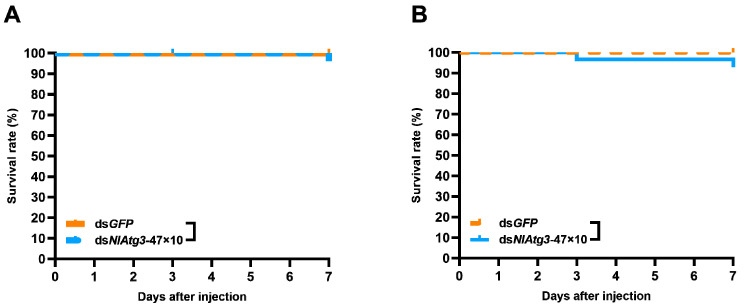
Effects of ds*NlAtg3*-47×10 injection on *D. sulfureus* and *T. chinensis* survival. Injection of ds*GFP* is used for controls. (**A**) Effect of ds*NlAtg3*-47×10 injection on *D. sulfureus* survival. (**B**) Effect of ds*NlAtg3*-47×10 injection on *T. chinensis* survival. The comparison of the survival curve was conducted by the Log-rank test. No asterisks indicate no significant differences among the comparisons.

The fifth-instar *N. lugens* nymphs were injected with 250 ng of ds*NlAtg3*-47×10, and 24 h later were fed to its natural enemies *D. sulfureus* and *T. chinensis*. After one week of feeding, it was found that no *D. sulfureus* had died in the ds*NlAtg3*-47×10 group (Figure 8A). The survival rate of the *T. chinensis* in the ds*NlAtg3*-47×10 group was only slightly decreased, with no significant difference found between the two groups (Figure 8B). This suggests that ds*NlAtg3*-47×10 has no obvious effect on the survival of its natural enemies *D. sulfureus* and *T. chinensis* through food chain transmission. These results suggest that ds*NlAtg3*-47×10 targeting against *N. lugens* is safe for the natural enemies *D. sulfureus* and *T. chinensis*.

## 4. Discussion

In this study, an autophagy-related gene, *NlAtg3*, was used to synthesize dsRNA fragments with different lengths and sequence compositions. Among the three dsRNA fragments (ds*NlAtg3-*474×1, ds*NlAtg3*-138×3, ds*NlAtg3*-47×10), the ds*NlAtg3*-47×10 fragment was proved to be specific and efficient against *N. lugens*, but was safe for non-target organisms, including two natural enemies (*D. sulfureus* and *T. chinensis*) of *N. lugens*. Therefore, this study establishes a foundation for the subsequent construction and application of rice-mediated RNA interference systems for *N. lugens*.

Previous RNAi experiments on potato beetles showed that the interference efficiency of 200–700 bp dsRNA was higher than that of 60 bp dsRNA in a feeding experiment, but there was no significant difference between dsRNA fragments with length varying from 200 bp to 700 bp [27]. Our work also supports that long dsRNA fragments (ds*NlAtg3*-474×1 and ds*NlAtg3*-138×3) covering a larger area of the target sequence achieved higher RNAi efficiency than that of the ds*NlAtg3*-47×10 fragment, which only covers a 47 bp area of the target sequence. In addition, transfection of dsRNA ranging from 21 to 592 bp in length resulted in effective target gene silencing in *Drosophila melanogaster* S2-cells, irrespective of the dsRNA length. In contrast, uptake of dsRNAs added to the medium was clearly length-dependent. Importantly, a diverse pool of short siRNAs (enzymatically generated 21-mer siRNAs) failed to enter S2 cells, and did not result in any significant silencing [18]. Currently, the number of 21-mer matches is considered a critical factor in the function of dsRNA transfected in cells, especially in its effects on non-target organisms [28,29]. In this study, the specific dsRNA fragment ds*NlAtg3*-47×10 showed a perfect interference effect on the target pest *N. lugens*, but did not cause significant lethality, neither to the closely related species *S. furcifera*, nor to the natural enemies *D. sulfureus* and *T. chinensis*. For ds*NlAtg3*-47×10 of *N. lugens*, the longest consecutive identical bases in *S. furcifera*, *D. melanogaster*, and *T. chinensis* were 12, 5, and 3, respectively, far fewer than 21 bp (Figure 3C). Therefore, by reducing the longest consecutive identical sequence in the dsRNA sequence, it is possible to avoid harming non-target organisms. Similarly, when conducting a non-target insect risk assessment on the *N. lugens* natural enemy species *C. lividipennis*, it was revealed that three dsRNA segments (ds*NlNa*, ds*NlAup5*, and ds*NlvATP-A*) specifically against *NlNa*, *NlAup5*, and *NlvATP-A* exhibited significant lethality towards *N. lugens*. However, when fed at high concentrations (10×), the survival rate, the spawning rate and adult survival time of non-target *C. lividipennis* did not show a significant impact [30]. It should be noted that very short dsRNA fragments may not be a good approach to reducing the non-target effect. They may bring other issues, for example, lost function for different populations with SNPs, or binding to unspecified genes in the target species and non-target species.

The evaluation criteria for non-target organism effects are important in studies on RNAi risk assessment. The selection of target genes, the dsRNA design, and the evaluation and investigation of the effects on non-target organisms can partly reduce the risk posed by RNAi-based biopesticides [31]. Tan et al. showed that no adverse effects on the growth and development were observed in either *Apis mellifera* L. larvae or adults at long and high *DvSnf7* dsRNA exposure levels [32]. Castellanos et al. demonstrated that feeding the parasitoid wasp *Telenomus*. *podisi* with wasp-specific dsRNA targeting the *vATPase A* and *actin-2* genes led to high mortality, demonstrating that dietary RNAi is functional in *T*. *podisi*. When feeding *T*. *podisi* with its host *Euschistus heros*-specific dsRNA targeting the same genes, no lethal or sublethal effects were observed [33]. Therefore, by reasonably designing and synthesizing suitable dsRNA, it is possible to acquire specific siRNA fragments and effectively improve the specificity and reduce the risk [33]. In future work, we will add some further comparisons with studies on other similar species, and above all, further investigate the effects on more non-target organisms.

Currently, transgenic plants expressing dsRNA have made significant breakthroughs in the management of insect pests. Three genes (*NlHT1*, *Nlcar* and *Nltry*) highly expressed in the midgut of *N*. *lugens* were used to develop dsRNA constructs for transforming rice, and some of the transcribed dsRNA was processed to siRNAs in the transgenic lines. When *N*. *lugens* fed on rice plants expressing dsRNA, the mRNA levels of the targeted genes in the midgut were reduced [10]. It has also been reported that rice plants with *osa-miR162a* overexpression had enhanced resistance to *N. lugens* and reduced the reproductive capacity of adults [11]. Some candidate corn varieties expressing the dsRNA of *DvvSnf7*, *Cry3Bb1* and *Cry34Ab1*/*Cry35Ab1* genes have been shown to cause significant mortality of western corn rootworm via the siRNA signaling pathway [8,9]. During a risk assessment of siRNA-mediated IRGE rice, it was found that the IRGE rice targeting the *dib* 3′ untranslated sequence had significant resistance to its target pest *C. Suppressalis*, but was sensitive to non-target organisms, and the impact on *A. mellifera* could be ignored [34]. When Baum et al. fed western corn rootworm (WCR) with transgenic corn plants which express WCR dsRNAs, there was a significant reduction in WCR feeding damage [35]. Therefore, RNAi-based, genetically engineered insect-resistant rice carrying species-specific dsRNA like ds*NlAtg3*-47×10, is expected to have good application potential.

## Data Availability

The raw data supporting the conclusions of this article will be made available by the authors on request.

## Supplementary Materials

The following supporting information can be downloaded at: https://www.mdpi.com/article/10.3390/insects16020225/s1.
